# Climbing the Ranks: A Study of Firefighter Health Disparities

**DOI:** 10.3390/healthcare12020227

**Published:** 2024-01-16

**Authors:** McKenzie M. Hare, Kealey J. Wohlgemuth, Alex Jesko, Michael J. Conner, Vanessa Frost-Piedrahita, Jacob A. Mota

**Affiliations:** 1 Neuromuscular and Occupational Performance Laboratory, Department of Kinesiology and Sport Management, Texas Tech University, Lubbock, TX 79409, USA; mckenzie.hare@ttu.edu (M.M.H.); kealey.wohlgemuth@ttu.edu (K.J.W.); 2 Front Line Mobile Health, Georgetown, TX 76048, USAmike@frontlinemobilehealth.com (M.J.C.); 3 Travis County Emergency Services, District #2, Pflugerville, TX 78660, USA; vfrost@pflugervillefire.org

**Keywords:** obesity, aerobic capacity, occupational performance, tactical, first responders

## Abstract

The fire service command structure encompasses recruit, incumbent firefighter, and officer positions. The purpose of this study was to quantify the effect of rank (recruits, incumbent firefighters, and officers) on health and physical ability characteristics within the fire service. Retrospective data from thirty-seven recruits (age = 29 ± 5 yrs, BMI = 26.5 ± 2.3 kg/m^2^); eighty-two incumbent firefighters (age = 30 ± 7 yrs, BMI = 28.8 ± 4.3 kg/m^2^); and forty-one officers (age = 41 ± 6 yrs, BMI = 28.6 ± 4.3 kg/m^2^) from a single department were used. Participants completed body composition tests (i.e., body fat percentage [%BF] and body mass index [BMI]), an air consumption test (ACT), and cardiopulmonary exercise testing. The ACT consisted of 10 standardized tasks. Five separate one-way analyses of co-variance (ANCOVA) were calculated, accounting for age. Partial eta squared statistics were calculated and Bonferroni-corrected post-hoc analyses were employed. The results demonstrated a significant effect of rank on %BF (*F* = 9.61, *p* < 0.001, η^2^ = 0.10); BMI (*F* = 3.45, *p* = 0.02, η^2^ = 0.05); relative VO_2MAX_ (*F* = 12.52, *p* < 0.001; η^2^ = 0.11); and HR_MAX_ (*F* = 18.89, *p* < 0.001, η^2^ = 0.03), but not on ACT time (*F* = 0.71, *p* = 0.55, η^2^ = 0.01). These outcomes suggest there are variations in anthropometric and physiological metrics of health across firefighter ranks. Administrators should be aware how these markers of health may vary across firefighter ranks.

## 1. Introduction

The fire service is known to implement a command structure that is composed of various leadership roles or ranks. Generally, firefighter ranks can be grouped into one of three categories: recruit, firefighter, and officer. Recruit firefighters encompass individuals who are new to the fire service and are actively participating in a firefighter training academy [1]. Though specifics vary across the country, recruit firefighters generally attend a structured fire training academy five days a week (Monday, Tuesday, Wednesday, Thursday, and Friday) from 8:00 a.m. to 5:00 p.m. The academy typically consists of both emergency medical response training (i.e., basic life support, advanced life support, and paramedic) and occupational specific training (i.e., forcible entry, extrication, and technical rescue). Recruits are typically considered employees while they are enrolled in the training academy, but do not respond to emergency calls or work at a fire station. Those with the rank of firefighter are individuals who have completed the firefighter academy training and work part- or full-time as an incumbent firefighter [2,3]. Firefighters work Kelly-style shifts (i.e., 24 h on/48 h off) at a fire station where they respond to emergencies as necessary. Officers include persons who hold leadership positions within the fire service (engineer, lieutenant, captain, battalion chief, etc.) [2,3]. Officers are required to direct elements of the fire ground or emergency scene in order to best utilize available assets (i.e., engine, ladder, or tanker companies) to accomplish their rescue, medical, or fire objective. Additionally, officers are responsible for their junior counterparts (i.e., incumbent firefighters), by leading training exercises and direction on the fireground, for example [2,3].

Regardless of rank, all fire personnel are required to perform varying strenuous tasks (i.e., stair climb, hoist, forcible entry, hose advance, and victim rescue) in order to execute the responsibilities of the occupation. In addition to performing physically demanding tasks, firefighters are required to wear personal protective equipment consisting of boots; bunker gear; a helmet; and a self-contained breathing apparatus, increasing the individual’s load and further adding to the strain of their tasks. Due to the varying demands required of the occupation, sufficient muscular strength [4,5], muscular power [4], and aerobic capacity [6] are necessary for successfully completing occupational performance tasks within the fire service. Unfortunately, it has been reported that many firefighters frequently do not meet the minimum physiological requirements of the job. Previous work has reported aerobic capacity is required to be greater than 38 mL/kg/min in order to successfully complete occupation-specific testing [7]. Conversely, the International Association of Firefighters and the International Association of Fire Chiefs have recommended all firefighters maintain an aerobic capacity of greater than 42 mL/kg/min [8]. The degree to which individual firefighter ranks may or may not meet this threshold is not yet understood.

While fire personnel are required to perform physically difficult tasks, firefighters are at greater risk of cardiovascular disease compared to the general population [9,10]. Cardiovascular disease is the most frequent mechanism of line-of-duty deaths in firefighters [11,12] with 45% of on-duty deaths caused by cardiovascular events [13]. Furthermore, recent work has suggested that subclinical cardiac disfunction may be linked to various cardiometabolic risk factors within the fire service [10]. Obesity is a major comorbidity in the development of cardiovascular disease, and is linked to mortality [14]. Subsequently, obesity is a concern among firefighters [15,16], with an increase in prevalence over time in the fire service [17,18]. The concern of obesity is not limited to its association with cardiovascular disease, as previous work has demonstrated a relationship between increased body mass index and lower cardiorespiratory capacity (i.e., estimated maximum oxygen consumption) [19]. This further suggests obesity is not only a risk factor for cardiovascular disease, but related to poor aerobic performance. Within the fire service human performance literature, there is a known association between simulated job tasks (i.e., forcible entry, victim rescue, and stair climb) and time taken to complete a 1.5 mile run [6]. Specifically, this relationship suggests that cardiorespiratory capacity is a valuable performance metric for the demands of firefighting. In addition, the fire service is comprised of individuals varying in status (i.e., rank), beginning as recruits, and then becoming incumbent firefighters and potentially officers, although the health and physical capabilities of firefighters across ranks in the fire service has yet to be thoroughly assessed. Therefore, the purpose of this study was to quantify the effect of rank (recruits, incumbent firefighters, and officers) on health and physical ability characteristics within the fire service. It was hypothesized that there would be a difference in health and physical ability characteristics across firefighter ranks.

## 2. Materials and Methods

### 2.1. Participants

Thirty-seven firefighter recruits (mean ± standard deviation [SD]: age = 29 ± 5 yrs, body mass index = 26.5 ± 2.3 kg/m^2^; 5 females); eighty-two incumbent firefighters (age = 30 ± 7 yrs, body mass index = 28.8 ± 4.4 kg/m^2^; 6 females); and forty-one officers (i.e., engineer, lieutenant, captain, battalion chief; age = 41 ± 6 yrs, body mass index = 28.6 ± 4.3 kg/m^2^; 1 female) volunteered to participate in this study. All participants (*n* = 160; 12 females) in this study were from a single fire department in the state of Texas within the United States of America. This study was granted ethical approval by the University Institutional Review Board (#2022-1009).

### 2.2. Experimental Design

Retrospective data were collected following a single fire department’s annual physical screening during 2022. Specifically, participants completed demographic questionnaires followed by body composition assessments. In addition, participants completed two separate physical ability tasks. In January of 2022 participants performed a maximal-effort cycle-based graded exercise test for the determination of maximal oxygen consumption and maximal exercise heart rate. On a separate occasion, in March of 2022, they were instructed to perform an air consumption test while in full bunker gear (i.e., turnout coat, pants, helmet, and self-contained breathing apparatus).

### 2.3. Anthropometrics and Body Composition

Height (m) and body mass (kg) were measured using a calibrated clinical stadiometer and scale, respectively. Anthropometric data were utilized for determining participant body mass index (body mass index = mass (kg)/height (m^2^)). Body mass index classifications can be categorized as normal weight = 18.5–24.9 kg/m^2^; overweight = 25.0–29.9 kg/m^2^; obese class I = 30.0–34.9 kg/m^2^; obese class II = 35.0–39.9 kg/m^2^; and obese class III > 40.0 kg/m^2^ [20]. Additional assessments consisted of a multifrequency bioelectrical impedance analysis scan (InBody 570, InBody USA, Cerritos, CA, USA) for the estimation of body fat percentage [21]. Prior to the multifrequency bioelectrical impedance analysis assessment, participants wore physical training attire, consisting of shorts and a t-shirt, and were instructed to remove shoes, socks, jewelry, and metals. For the multifrequency bioelectrical impedance analysis assessment, participants were asked to stand as still as possible on the electrodes while keeping their hands firmly grasped to the handles with their thumbs on the electrodes and arms away from the body. The body composition assessment was completed following a minimum three-hour fast and all participants were euhydrated during the assessment.

### 2.4. Cardiopulmonary Exercise Testing

The cardiopulmonary exercise testing consisted of a maximal-effort cycle-based graded exercise test. During the task the maximal heart rate (HR_MAX_) was recorded utilizing a 12-lead electrocardiogram (ECG) in beats per minute (bpm). The task was performed on a cycle ergometer (Lode Corival, Lode B.V., Groningen, The Netherlands) and used to determine maximal oxygen consumption (VO_2MAX_). The cardiopulmonary exercise test began with a 1 min warm-up where participants were instructed to cycle at 70 repetitions per minute (rpm). Immediately following the warm-up, the power output was increased by 30 watts. Power output then continued to increase by 30 watts every minute until the participant was no longer able to maintain the power output. During the task, respiratory gases were monitored with open-circuit spirometry using a calibrated metabolic cart (Ultima Cardi02, MGC Diagnostics, St. Paul, MN, USA). Analysis was completed using a 6-breath rolling average with VO_2MAX_ as the highest oxygen consumption during the exercise test.

### 2.5. Air Consumption Test

All participants completed the air consumption test in full bunker gear (i.e., turnout coat, pants, helmet, and self-contained breathing apparatus). The air consumption test was based on the Forces Firefighter Physical Fitness Maintenance Evaluation [22]. Participants were assessed for the time taken to complete all 10 of a set of standardized occupation-specific tasks. Each task was located 50 to 100 feet apart, with the tasks set up as a circuit. Time taken to complete all tasks in a single bout was recorded in minutes with a stopwatch. Unlike traditional aerobic capacity testing (i.e., VO_2MAX_), the air consumption test examines aerobic capacity by monitoring the air consumed out of a self-contained breathing apparatus (standard equipment for firefighters) during the completion of the aforementioned procedures. Therefore, this task mimics the real-world requirements of firefighters (i.e., performing occupation-specific tasks while using a self-contained breathing apparatus). Tasks are described in the order in which they were performed.

1. One-arm hose carry: in one hand, the participant held a rope handle with 50 ft (15.2 m) of fire hose weighing 16.5 kg (36.4 lb), then carried it 50 ft (15.2 m), before switching hands and carrying the fire hose the same distance. Following this event, the participant walked 50 ft (15.2 m) to the next event.
2. Ladder carry and raise: the participant lifted a 12 ft (3.6 m) aluminum ladder, carried it 50 ft (15.2 m), then secured it against a wall. Following the event, the participant walked 50 ft (15.2 m) to the next event.
3. Hose drag: the participant lifted and placed a hose nozzle over their preferred shoulder, then dragged two lengths (50 ft; 15.2 m) of a hose 1.75 in diameter a total distance of 100 ft (30.6 m). Following the event, the participant walked 50 ft (15.2 m) to the next event.
4. Ladder climb I: the participant climbed 10 rungs (12 ft; 3.6 m) up and down a 24 ft (7.3 m) ladder, three times. Following the event, the participant walked 100 ft (30.6 m) to the next event.
5. Sled pull: using a hand-over-hand movement, the stationary participant pulled a rope attached to a weighted sled 50 ft (15.2 m), then walked 50 ft (15.2 m) and repeated the pull. Following the event, the participant walked 50 ft (15.2 m) to the next event.
6. Forcible entry: the participant used a 4.54 kg (10 lb) sledgehammer to hit a target on a mechanical apparatus. Following the event, the participant walked 50 ft (15.2 m) to the next event.
7. Victim rescue: while walking backward the participant dragged a 79.8 kg (175.9 lb) mannequin a distance of 85 ft (25.9 m). Following the event, the participant walked 50 ft (15.2 m) to the next event.
8. Ladder climb II: the participant climbed 10 rungs (12 ft; 3.6 m) up and down, two times, on a 24 ft (7.3 m) ladder. Following the event, the participant walked 100 ft (30.6 m) to the next event.
9. Ladder lower: the participant lowered and carried a 12 ft (3.6 m) aluminum ladder 50 ft (15.2 m). Following the event, the participant walked 50 ft (15.2 m) to the next event.
10. Equipment carry: the participant picked up and carried a bar with weight plates and collars weighing a total 36.3 kg (80 lb), a distance totaling 100 ft (30.5 m). The test concluded when the participant finished the carry. Lowering the equipment was not a part of the test.

### 2.6. Statistical Analysis

Five separate one-way analyses of co-variance (ANCOVA) were calculated to examine differences in health (i.e., body mass index, and body fat percentage) and physical ability (i.e., air consumption test time, VO_2MAX_, and HR_MAX_) metrics across firefighter ranks (i.e., recruit, incumbent firefighter, and officer). Each model accounted for age as a co-variate by including it as an additional independent, continuous variable within each model, respectively. The premise of the aforementioned ANCOVA model allowed for the interpretation of the main effects after each rank was adjusted to the same mean age. Additionally, partial eta squared (η^2^) statistics were calculated to serve as a measure of effect size. Bonferroni-corrected post-hoc analyses were utilized in the event of a significant main effect. All data were presented as unadjusted mean ± standard deviation (SD) and analyzed in R (Version 4.2.1 “Funny-Looking Kid”, R Foundation for Statistical Computing, Vienna, Austria) using the integrated development environment RStudio (Version 2022.07.1-554 “Spotted Wakerobin” for MacOS, PBC, Boston, MA, USA). Within RStudio, multiple supporting packages were utilized including tidyverse [23], MASS [24], and emmeans [25]. The alpha level was set a priori to 0.05 to determine statistical significance.

## 3. Results

The demographic characteristics of the total sample (*n* = 160; 12 females) categorized by recruits, incumbent firefighters, and officers, along with associated pairwise comparisons, are presented in Table 1. After accounting for age, the outcomes of the five separate one-way analyses of co-variance demonstrated a statistically significant effect of rank on body mass index (*F* = 3.45, *p* = 0.02, η^2^ = 0.05); body fat percentage (*F* = 9.61, *p* < 0.001, η^2^ = 0.10); relative VO_2MAX_ (*F* = 12.52, *p* < 0.001, η^2^ = 0.11); and HR_MAX_ (*F* = 18.89, *p* < 0.001, η^2^ = 0.03). However, air consumption test time (*F* = 0.71, *p* = 0.55, η^2^ = 0.01) did not significantly differ across ranks (Table 2). Following Bonferroni post-hoc adjustments, recruits demonstrated significantly lower body mass index (26.46 ± 2.33 kg/m^2^) than the incumbent firefighters (28.77 ± 4.35 kg/m^2^; *p* = 0.02), but not the officers (28.63 ± 4.33 kg/m^2^; *p* = 0.32). Recruits had a significantly lower body fat percentage (17.52 ± 4.99%) than the incumbent firefighters (24.07 ± 8.46%; *p* < 0.001), but not the officers (23.90 ± 7.30%; *p* = 0.18). Additionally, recruits demonstrated significantly greater relative VO_2MAX_ (40.39 ± 4.63 mL/kg/min) compared to the incumbent firefighters (34.48 ± 6.37 mL/kg/min; *p* < 0.001), but not the officers (34.19 ± 6.46 mL/kg/min; *p* = 0.06). Lastly, the officers displayed significantly greater HR_MAX_ than the incumbent firefighters (176.77 ± 11.30 bpm; *p* = 0.05), but not the recruits (181.00 ± 9.29 bpm; *p* = 0.63). There were no statistically significant comparisons for HR_MAX_ between the recruits and the incumbent firefighters (173.88 ± 10.90 bpm; *p* = 0.31).

## 4. Discussion

Fire personnel of all ranks are required to perform critical and essential job tasks which may be considered physically demanding [26]. The present investigation is the first to determine the influence of firefighter rank (i.e., recruit, incumbent firefighter, and officer) on multiple health characteristics (i.e., body mass index and body fat percentage). The purpose of this study was to quantify the effect of rank (recruits, incumbent firefighters, and officers) on health and physical ability characteristics within the fire service. The results of the present study suggest body mass index, body fat percentage, and relative VO_2MAX_ demonstrate statistically significant differences across rank. Specifically, these findings suggest lower body mass index and body fat percentage in recruits compared to incumbent firefighters. In addition, the results revealed greater VO_2MAX_ in recruits compared to incumbent firefighters. These findings support previous reports which highlight the growing problem of obesity and poor cardiovascular health within the fire service [16,27], with the present study additionally reporting the novel effect of rank on the prevalence of poor health metrics.

### 4.1. Firefighter Health

Despite firefighters needing to perform demanding occupational specific tasks, obesity is a common health concern in the fire service [9,15,16]. The present study suggests recruit firefighters demonstrate superior body composition (i.e., body mass index and body fat percentage) compared to incumbent firefighters. Although recruits and incumbent firefighters are required to complete the same occupational tasks, recruits are not yet full-time firefighters; instead, as part of the training process, they are typically in an instructional setting from 8:00 am to 5:00 pm and do not respond to emergency calls. Conversely, incumbent firefighters and officers work in Kelly-style shifts and are required to perform many duties, including emergency response. Given the substantial overlap in occupational duties across the three ranks, it is likely that age is one of many factors to be considered. In support of our present findings, Bond et al. [27] reported that with advancement in age, male firefighters increased their body mass by 0.42 kg, body mass index by 0.13 kg/m^2^, and body fat percentage by 0.18% per year [27]. Furthermore, Davis et al. [17] reported in a sample of male and female firefighters an increase in body mass of 0.47 kg, body mass index of 0.14 kg/m^2^, and body fat percentage of 0.31% per year of employment. Collectively, these works [17,27] suggest that firefighter metrics of body composition may decline longitudinally with age; however, this alone may not provide insight into the effect of firefighter rank on health. While no studies have directly compared recruits to incumbent workers, previous works have reported recruits, on average, to have a body mass index of approximately 26.6 kg/m^2^ [28,29], while separate investigations have reported male career firefighters’ body mass index to range from 28.1–33.1 kg/m^2^ [9,15,30,31]. This increase in body mass index between recruits and incumbent firefighters suggests that a decline in body composition may be present and simultaneously supports the outcomes of the present investigation. To the knowledge of the authors, no previous study has investigated differences in health across firefighter ranks (i.e., recruit, incumbent firefighter, and officer). While it may be that firefighter rank is related to age (e.g., older firefighters may become officers, while younger firefighters may be recruits), and therefore it should be expected that health characteristics would change over a lifespan; it is important to note that young officers, especially lieutenants and captains, are commonplace in the fire service. Nonetheless, the present investigation adjusted for age within each ANCOVA model, as described earlier. As such, the present study indicates rank may have an effect on health, after adjusting for age, specifically for body mass index (recruits = 26.46 ± 2.33 kg/m^2^, incumbent firefighters = 28.77 ± 4.35 kg/m^2^, officers = 28.63 ± 4.33 kg/m^2^), and body fat percentage (recruits = 17.52 ± 4.99%, incumbent firefighters = 24.07 ± 8.46%, officers = 23.90 ± 7.30%). Despite more senior ranking firefighters needing to physically qualify to perform duties or physical abilities tests in a similar capacity to their younger counterparts, their health and chronic disease risk significantly differs. Fire administrators are encouraged to carefully examine the health of their officers. In the event that officers within the fire service are able to improve their individual health, junior firefighters or recruits may be inclined to further improve their own health by following the direction and attitudes of their leadership.

### 4.2. Physical Ability in Firefighters

Firefighters who possess greater cardiovascular endurance may be better able to perform the physical demands required of the job [6]. Willford et al. [6] found that greater cardiovascular endurance in firefighters, (i.e., the 1.5 mile run time), was related to improved job performance (i.e., the stair climb, hoist, and forcible entry). In addition, previous work by Baur et al. [19] reported a significant relationship between body mass index and cardiorespiratory endurance, which may additionally influence occupation-specific performance [6]. In support of this finding, the present study suggests that firefighters who display greater VO_2MAX_, also exhibit lower body mass index and body fat percentage. Furthermore, the present findings expand on this relationship by revealing greater VO_2MAX_, lower body mass index and lower body fat percentage for recruits compared to incumbent firefighters. A systematic review by Ras et al. [32] has suggested as firefighters increase in age, their performance on various tasks (i.e., the stair climb, or victim drag) significantly decreases [32]. Conversely, the current study suggests regardless of rank, firefighters perform similarly on occupation-specific tasks (i.e., the air consumption test time), yet recruit firefighters display greater aerobic capacity (VO_2MAX_) than incumbent firefighters. It is unsurprising that recruits display more favorable aerobic capacity outcomes. Cornell et al. [28] examined health and fitness changes in recruits immediately following the training academy and following the initial five months as an incumbent firefighter. The authors report incumbent firefighters (~5 months following training academy) having significant decrements in aerobic capacity compared to as a recruit [28]. The present study investigated the influence of firefighter rank on occupational performance (i.e., the air consumption test). Similar to the aforementioned study [28], the present study displayed differences in physical ability metrics between recruit firefighters and their senior counterparts, further suggesting recruit firefighters demonstrate better aerobic capacity and may be more physically prepared for the demands of the occupation. Fire service administrators and policy makers may consider making standards that vary across the career of a firefighter to promote high physical capability standards no matter if a recruit, incumbent firefighter, or officer. Future studies may examine longitudinal changes in health and performance metrics assessing junior firefighters across their career in lieu of cross-sectional study designs such as that used in the present investigation.

### 4.3. Limitations

Although in the present study the sample included female firefighters, the inclusion was limited to 12 female firefighters. As such, this study did not investigate potential sex differences and the results may not be generalizable to female firefighters. The air consumption test is frequently utilized in the fire service for examining firefighter occupational performance, yet there is limited research investigating air consumption test in the literature. Thus, differences in air consumption test style or tasks within the assessment may influence outcome measures of future studies. Additionally, due to logistical constraints, cardiopulmonary exercise testing and the air consumption test were not assessed within the same data collection session which may have allowed for individuals’ aerobic capacity to change. While this time effect may have influenced the relationship between aerobic capacity (VO_2MAX_) and the air consumption test, it is unlikely to have influenced the primary research question in this study. Finally, the present dataset is cross-sectional in nature and did not have psychosocial motivator factors (i.e., motivation, habits, and goals); future studies may wish to longitudinally address this to better understand what additional factors may change across ranks within the fire service.

## 5. Conclusions

The purpose of this study was to determine differences in health and physical ability across firefighter ranks. These outcomes suggest variations in anthropometric and physiological metrics across recruits, incumbent firefighters, and officers in the fire service. Although it was determined that air consumption test time did not differ between ranks, the present findings suggest greater aerobic capacity and more favorable body composition was found in recruits compared to incumbent firefighters and officers. Furthermore, these findings collectively suggest that while senior firefighters may be able to perform the physical demands required of the occupation their health and chronic disease risk may be disadvantageously positioned, compared to their junior counterparts who may in better health. Future work may aim to obtain a nationally representative sample of firefighters to appropriately designate health and physical ability standards for each rank within the fire service. In the meantime, fire administrators should be aware of the possible discrepancies in health and physical ability across firefighter ranks and further implement strategies to reduce chronic health concerns.

## Figures and Tables

**Table 1:** Demographic characteristics (mean ± standard deviation [SD]) of the 160 firefighters (12 females); categorized by recruits (*n* = 37), incumbent firefighters (*n* = 82), and officers (*n* = 41). The table reports age in years; body mass index (BMI) in kg/m^2^; body fat as a percentage (%BF) of total mass; maximal aerobic capacity (VO_2MAX_) in mL/kg/min; maximal heart rate (HR_MAX_) in beats per minute (bpm); and air consumption test time (ACT Time) in minutes (min) for firefighters grouped by firefighter rank. Pairwise comparisons for each rank and associated health and physical ability characteristic were performed using the Bonferroni post-hoc comparison procedure. Comparisons which were deemed statistically significant are indicated below.

	Recruits (*n* = 37)	Incumbent Firefighters (*n* = 82)	Officers (*n* = 41)
Age (years)	29 ± 5	30 ± 7	41 ± 6
BMI (kg/m^2^)	26.46 ± 2.33 *	28.77 ± 4.35	28.63 ± 4.33
%BF (%)	17.52 ± 4.99 *	24.07 ± 8.46	23.90 ± 7.30
VO_2MAX_ (mL/kg/min)	40.39 ± 4.63 *	34.48 ± 6.37	34.19 ± 6.46
HR_MAX_ (bpm)	181.00 ± 9.29	176.77 ± 11.30	173.88 ± 10.90 ^†^
ACT Time (min)	6.91 ± 0.82	7.09 ± 0.71	7.10 ± 0.72

* Significant difference between recruits and incumbent firefighters. ^†^ Significant difference between officers and incumbent firefighters.

**Table 2:** Outcomes from the five one-way analyses of co-variance (*F*) to examine the effect of rank on body mass index (BMI); body fat percentage (%BF); relative aerobic capacity (VO_2MAX_); maximal heart rate (HR_MAX_); and air consumption test time (ACT Time) for 160 firefighters (12 females). All models accounted for age as a covariate. Partial eta squared (η^2^) statistics were used as a measure of effect size.

	*F*	*p*	η^2^
BMI (kg/m^2^)	3.45	0.02	0.05
%BF (%)	9.61	<0.001	0.10
VO_2MAX_ (mL/kg/min)	12.52	<0.001	0.11
HR_MAX_ (bpm)	18.89	<0.001	0.03
ACT Time (min)	0.71	0.55	0.01

## Data Availability

Data may be provided upon request.
